# Computer-aided diagnosis of pectus excavatum using CT images and deep learning methods

**DOI:** 10.1038/s41598-020-77361-y

**Published:** 2020-11-20

**Authors:** Lixuan Lai, Siqi Cai, Luyu Huang, Haiyu Zhou, Longhan Xie

**Affiliations:** 1 Shien-Ming Wu School of Intelligent Engineering, South China University of Technology, Guangzhou, 510640 China; 2 Division of Thoracic Surgery, Guangdong Lung Cancer Institute, Guangdong Provincial People’s Hospital and Guangdong Academy of Medical Sciences, Guangzhou, 510080 China; 3 School of Medicine, South China University of Technology, Guangzhou, 510640 China

**Keywords:** Biophysics, Diseases

## Abstract

Pectus excavatum (PE) is one of the most common chest wall defects. Accurate assessment of PE deformities is critical for effective surgical intervention. Index-based evaluations have become the standard for objectively estimating PE, however, these indexes cannot represent the whole information of chest CT images and may associated with significant error due to the individual differences. To overcome these limitations, this paper developed a computer-aided diagnosis (CAD) system based on the convolutional neural network (CNN) to automatically learn discriminative features and classify PE images. We also adopted block-wise fine-tuning methods based on the transfer learning strategy to reduce the potential risk of overfitting caused by limited data and experimentally explored the best fine-tuning degree. Our method achieved a high level of classification accuracy with 94.76% for PE diagnosis. Furthermore, we proposed a majority rule-based voting method to provide a comprehensively diagnostic results for each patient, which integrated the classification results of the whole thorax. The promising results support the feasibility of our proposed CNN-based CAD system for automatic PE diagnosis, which paves a way for comprehensive assessments of PE in clinics.

## Introduction

Pectus excavatum (PE) is one of the most common chest wall defects occurring once in every 400 newborns^1^. PE is a type of congenital malformation in which the sternum and thorax of the anterior thoracic wall are recessed inward relative to other anterior chest walls^2^. The deformity causes a funnel-like depression in the chest, which not only affects normal cardiovascular and respiratory functions, but also leads to exercise intolerance and physical limitations^3^. To repair the PE, surgical techniques are commonly used^4^.

Accurate measures of PE are critical for effective surgical interventions and many methods have been proposed to assess the severity of PE. To the best of our knowledge, Haller et al.^5^ firstly used medical images to quantify the degree of a chest wall deformity, which is referred as the Haller index (HI). The HI, which is defined as the widest transverse diameter of the internal chest divided by the distance between the anterior spine and posterior sternum, has been widely employed as the decision criterion for surgical correction^6^. Additionally, some approaches have been proposed to modify HI and improve the accuracy of PE diagnosis^7–10^. However, these assessment methods proposed in previous studies focused on some indexes which were selected based on clinic experiences to reflect the chest shape. Neither HI nor other modified indexes can represent the whole information of chest CT images. Due to individual differences in chest shape, existing indexes could hardly be applied as a universal diagnostic and assessment approach, especially in non-standard chest cases, such as narrow chest and asymmetric chest wall. To overcome this limitation, we proposed to use convolutional neural networks (CNN) to automatically learn representations from CT images and evaluate the PE deformities. To our knowledge, no previous study has evaluated the feasibility of using a CNN-based computer-aided diagnosis (CAD) system to assess the severity of PE deformities.

CAD is a computerized process that can assist radiologists in interpreting and diagnosing medical images and provide a second objective opinion to improve the clinical diagnostic results^11^. CNN is a deep learning method which has a natural advantage in utilizing the 2D structure of an image^12^. CNN-based CAD systems have been applied in many medical fields and have achieved promising results in the detection of lung nodules, breast lesions and colonic polyps^13–15^. Inspired by this, we investigate whether a CNN-based CAD system can be employed as an end-to-end classifier to automatically diagnose PE. Without any requirement for subjective and manual feature extraction processes, the proposed CNN model can automatically learn discriminative features from CT images and classify PE cases. In addition, it can overcome the diagnostic difficulty caused by the individual differences in PE and has excellent diagnostic performance in cases never seen.

In general, training CNN models requires large-scale annotated datasets, which are usually unavailable in medical fields due to the high manual labeling costs^16^. For small datasets, training the CNN models from scratch may require excellent computational power, sufficient memory resources and considerable time^16,17^. To address these issues, many researchers proposed the transfer learning strategy, that is, to utilize the CNN weights that are pretrained on large-scale public datasets to initialize the desired models^18^. However, the transferability from natural images to CT images with PE symptoms has not yet been explored in previous studies. In this study, we investigated whether transfer learning can be applied to a CT dataset of PE and explored the impact of transfer learning on the model’s performance.

Besides automatic assessment of PE using CT images, we have developed a majority rule-based voting method to provide comprehensive diagnoses for patients with PE. Existing approaches for PE diagnosis depend on one or a few images of patients, which may lead to incomplete understanding of the patient’s condition. Though images of the most serious deformation part of the thoracic wall usually can reflect the severity of PE, partial images cannot carry out the whole information. Based on the proposed automatic assessment method, we detected all images of the patient with high efficiency and took all images of the chest into consideration for a more comprehensive diagnosis of PE. In our CAD system, we received and predicted all images of each patient, and output the corresponding proportions of the three labels as a reference to assisting radiologists in diagnosis. We also adopted the majority rule-based voting strategy to draw a final comprehensive conclusion for the severity of each patient’s PE deformities.

There are threefold of this study. First, we introduced the CNN method to automatically and intelligently extract discriminative features from the CT images for assessing the severity of patients with PE. It is the first study to develop and evaluate a CNN-based CAD system for PE diagnosis. Second, we adopted the transfer learning strategy using the pretrained weights of the VGG16 and VGG19 networks and fine-tuning the top few layers block-wise to improve the classification performances on our dataset. Our proposed method achieved a high level of classification accuracy with 94.76% for PE diagnosis, which verified the feasibility of CNN models in PE diagnosis. Finally, we proposed a CAD system to integrate the classification results of various parts of the thorax and draw comprehensive diagnostic conclusions from the perspective of individual patients.

The remainder of this paper is organized as follows. “Materials and methods” describes the source and distribution of the dataset, the CNN model that applied the transfer learning strategy to classify the severity of PE deformities, and the method proposed to comprehensively evaluate the severity of PE deformities in patients. “Results” gives the details of the training experiment and shows our results, and “Discussion and conclusion” discusses our observations from the results and concludes the paper.

## Materials and methods

### Dataset

**Figure 1:**
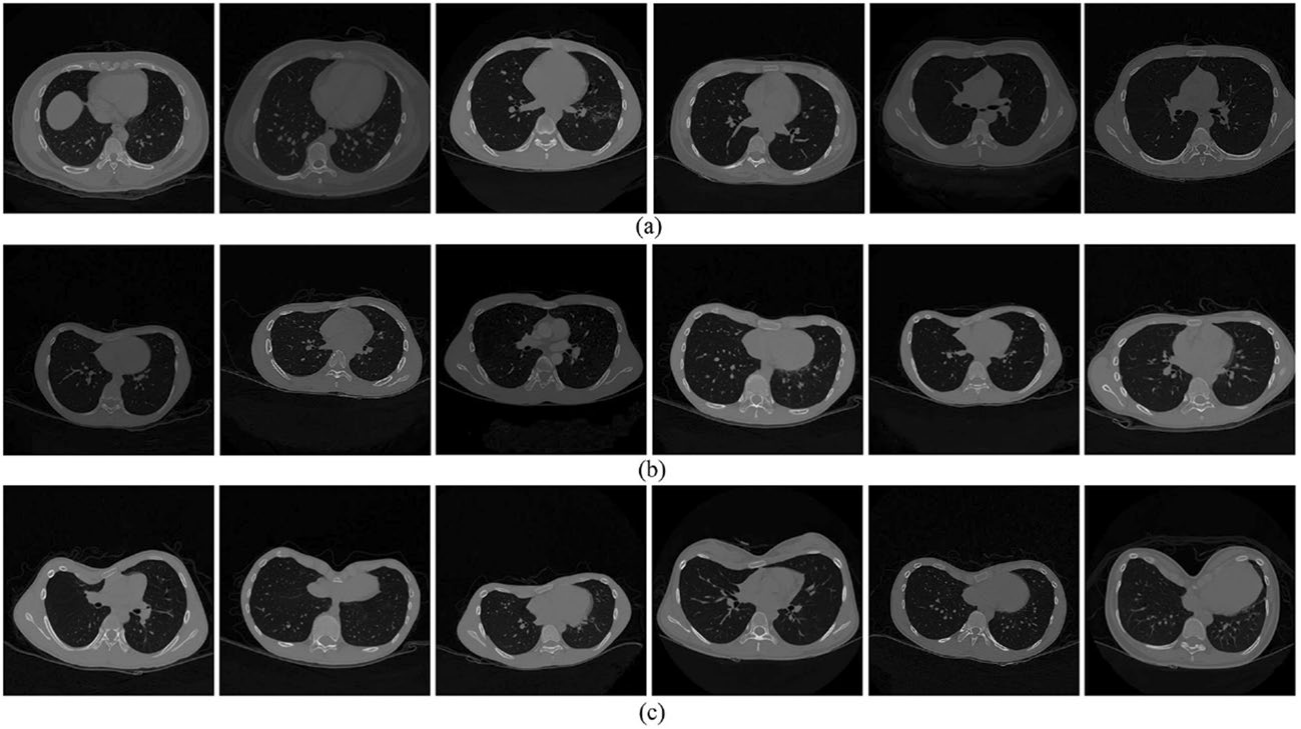
Samples of chest contours with different shapes. (**a**) Chests of normal shape. (**b**) Chests with mild PE. (**c**) Chests with severe PE.

Because there are no publicly available datasets on PE, we collected the dataset from 42 subjects at Guangdong General Hospital from 2010 to 2019 in this study. All participants received a traumatic chest CT routine scan on a Philips iCT 256 scanner (Philips Brilliance ICT 256 MDCT, Philips Healthcare, Best, The Netherlands). The scanned breast contour is saved in the form of CT images. Among these 42 subjects, there were 27 subjects with PE (22 males, age $15.5\pm 2.7$ years) and 15 healthy subjects (11 males, age $19.3 \pm 5.4$ years). All the images were labeled by experienced radiologists and divided into three categories, including normal, mild and severe, as shown in Fig. 1. It can be seen that there are individual differences among different subjects, which may be caused by the diversity of body shape, such as height and weight. Moreover, various kinds of human tissues are mixed in CT images, which may bring interference on sternum contour recognition.

In addition, the radiologists also marked a general label as the final diagnosis result for each participant based on the overall condition of the sternum outline. Table 1 shows the details of the dataset.

**Table 1:** Details of the dataset.

Chest shape	Number of subjects	Number of CT images
Normal	15	1176
Mild PE	15	1394
Severe PE	12	1133
Total	42	3703

The dataset consists of 3703 images from 42 subjects.

### Methodology

#### Preprocessing

##### Normalization

 Data preprocessing is performed to facilitate the subsequent training of the neural network. Neural networks are not very good at processing data with relatively large values or large differences because these issues are not conductive to the training of the neural network. They may cause a large gradient update of the neural network and thus the neural network would not converge. Therefore, it is necessary to normalize the data before neural network training. The normalization is usually performed to transform the input values of the CNN model and confine them to the same range. In this paper, we performed min-max normalization on each channel of the image, that is, in each channel we subtracted the minimum intensity value of the entire image from each pixel, and then divided the result by the difference between the maximum intensity value and the minimum intensity value so that any intensity value of the input image is in the range of [0–1]^19^. The computing process is as follows:

$$\begin{aligned} \begin{aligned} y_i=(x_i-min(x))/(max(x)-min(x)) \end{aligned} \end{aligned}$$

where $y_i$ is the normalized intensity value corresponding to the certain position $x_i$ (where $i=1,2,\ldots ,n$) and *max*(*x*) and *min*(*x*) are the maximum and minimum intensity value of the entire image, respectively. In addition, the original images have a size of $512\times 512$ pixels, which is unsuitable for most pretrained networks because they must receive a default smaller image size. Therefore, we resized the input images to $224\times 224$ to fully take advantage of the pretrained VGG networks by reducing the memory requirements.

##### Data augmentation

**Figure 2:**
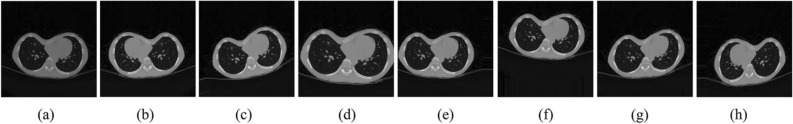
Examples of images processed via data augmentation. (**a**) The original image. (**b**) Horizontally flipped image. (**c**) Rotated image. (**d**) Zoomed image. (**e**) Horizontally shifted image. (**f**) Vertically shifted image. (**g**) Sheared image. (**h**) A sample image processed by all the transformation techniques.

Theoretically, the excellent performance of a deep neural network greatly relies on the scale of the labeled dataset. Unfortunately, large-scale labeled datasets are unavailable in some medical domains. Therefore, some techniques have been performed to prevent overfitting when training a neural network on small dataset, such as data augmentation. Data augmentation is a technique that adds noise or applies geometric transformations to existing pictures^17^. Geometric transformation is based on image manipulation processes, including flipping, cropping, rotation, color space transformation and so on. In this research, we performed a random rotation ranging from $0^{\circ }$ to $20^{\circ }$; a random horizontal and vertical shift of 25% of its total width and height, respectively; a zoom range between [0.8–1.2]; and a shear range of 0.2 (shown in Fig. 2). In addition, we randomly flipped the images horizontally and it did not change the image label.

#### Convolutional neural network architectures

A CNN is a type of deep learning network that evolved from the multilayer perceptron. It has been proven that a CNN can be effectively applied in areas such as image recognition and classification, object detection and natural language processing. Unlike the traditional multilayer perceptron that usually uses a fully connected network, a CNN performs well in the field of image processing due to its structural characteristics of local connectivity, shared weights and down sampling.

**Figure 3:**
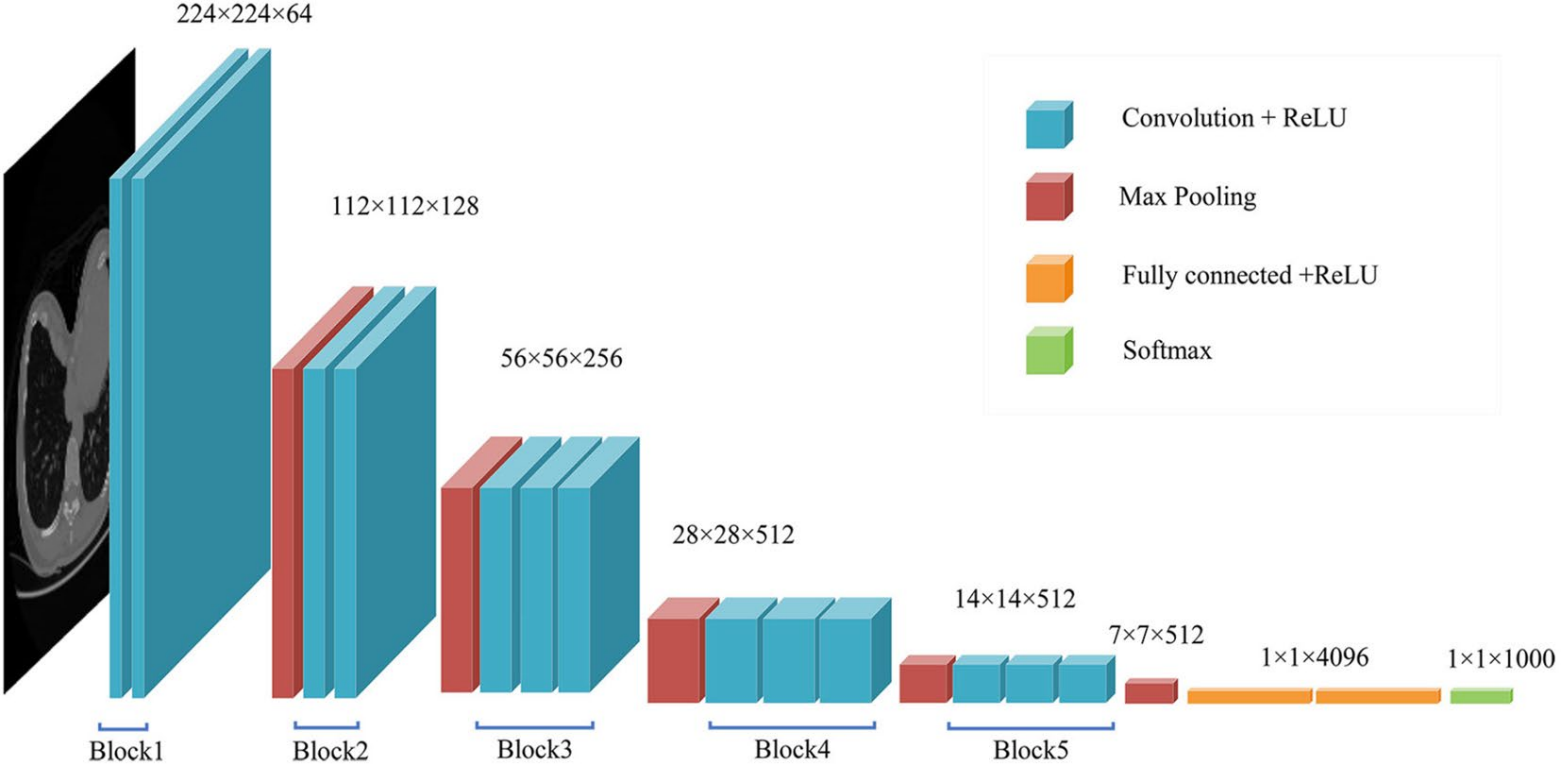
The architecture of the VGG16 network. It is composed of five convolutional blocks and three fully connected layers.

In this research, we introduced two state-of-art CNN architectures: VGG16 and VGG19. We utilized these CNN architectures and transferred the weights that are pretrained on ImageNet to achieve excellent classification performance on our own dataset. VGG16 is composed of 13 convolutional layers and 3 fully connected layers (shown in Fig. 3) while VGG 19 consists of 16 convolutional layers and 3 fully connected layers. They both contain 5 convolutional blocks, each followed by a pooling layer.

#### Transfer learning

In most cases, the size of an annotated dataset largely determines the performance of a CNN on an image classification task. It usually takes researchers many years to collect data and build a large-scale dataset. Access to large-scale public datasets is very helpful for researchers. However, in many medical fields, obtaining a large amount of labeled data can be very expensive due to the high manual labeling costs and scarcity of medical cases. When we train a CNN from scratch using a small dataset, we will most likely face the risk of overfitting. A model trained on limited data will lack generalizability, that is, the model will not have sufficient classification capabilities for many situations that have never been seen before. Under this circumstance, transfer learning is proposed to solve this dilemma.

Transfer learning is a machine learning technique that utilizes the knowledge gained from a certain field and then applies it to a new related field. Many researchers suggested that transfer learning has the potential to greatly improve a model’s generalization ability and make models more robust^20^. A neural network trained on a huge amount of data in a domain can be used to retrain a small sized dataset in another related but different domain to achieve the transfer of knowledge. In addition, it is proven by Zhou et al.^21^ that transfer learning from ImageNet to other datasets with limited scales can learn deeper models with better performance. Similar to the hypothesis in previous studies^18,22^, we assumed that although natural images differ greatly from medical images, pretrained weights on large-scale annotated datasets, such as ImageNet, can be transferred to small datasets.

There are two main types of transfer learning related to deep learning. (1) A CNN pretrained on large datasets can be used as a feature extractor^23^. We can load the pretrained model and extract the output of the specified layer as the feature vector, which can be used to train traditional machine learning classifiers, such as the logistic regression (LR) and support vector machine (SVM). (2) The fully connected layers of the pretrained model can be replaced with new fully connected layers and the pretrained weights can be fine-tuned to perform classification tasks on our dataset. We will introduce these two methods separately in the next two sections.

#### Feature extractor: a simple way to apply transfer learning

A CNN is usually used as an end-to-end image classifier, that is, when the model receives input images, forward propagation is performed throughout the network and the classification results are output at the end. In fact, any intermediate layer (such as an activation layer or a pooling layer) can be treated as the output end, and its output can be extracted as feature vectors. Subsequently, we can use these feature vectors to train a traditional machine learning model.

A simple way to implement transfer learning is to use a pretrained network as a feature extractor. First, we chose a CNN pretrained on ImageNet and removed the top layers, including the fully connected layers and SoftMax classifier. The rest of the pretrained network was treated as a feature extractor for a new dataset and could generate a feature vector when images are input. Second, we normalized the extracted features, and removed the unnecessary features via principal component analysis (PCA). PCA algorithm is a method that can reduce the data dimension while minimizing the loss of information. It is usually used for data preprocessing and data compression^24^. Finally, we trained a new classifier, such as the SVM, LR and decision tree (DT), using the normalized and selected feature vector. It is noted that this way can take advantage of the pretrained networks to easily extract features and overcome the complex and time-consuming defects of the traditional feature extraction method. In addition, the training process has no high requirements on computing power and is relatively simple compared to full deep learning training.

#### Block-wise fine-tuning pretrained network

In this research, we build our models using transfer learning and a fine-tuning strategy in this way.

First, we kept the original structure except the fully connected layers and inserted the new fully connected layers behind it. The original architecture of the pretrained CNN was designed for the ImageNet dataset that includes 1000 categories^25^. Therefore, we modified the fully connected layers and built a new SoftMax classifier with three outputs for three categories: normal, mild and severe.

Second, we froze the weights of all layers except the fully connected layers, and randomly initialized the weights of the new fully connected layers. Then, we trained the model on our dataset with a small learning rate. The initial parameters of the convolution layers transferred from the pretrained network contain a large number of useful convolution filters with powerful feature discrimination capabilities. However, the weights of the newly added fully connected layers are completely new and random, and the weight update may damage the feature extraction capability of the aforementioned pretrained convolution layers due to the back propagation in the whole network. Therefore, it is necessary to freeze the pretrained convolution layers. This enables back propagation to be restricted among the fully connected layers when the fully connected layers are trained and forward propagation is performed throughout the whole network.

**Figure 4:**
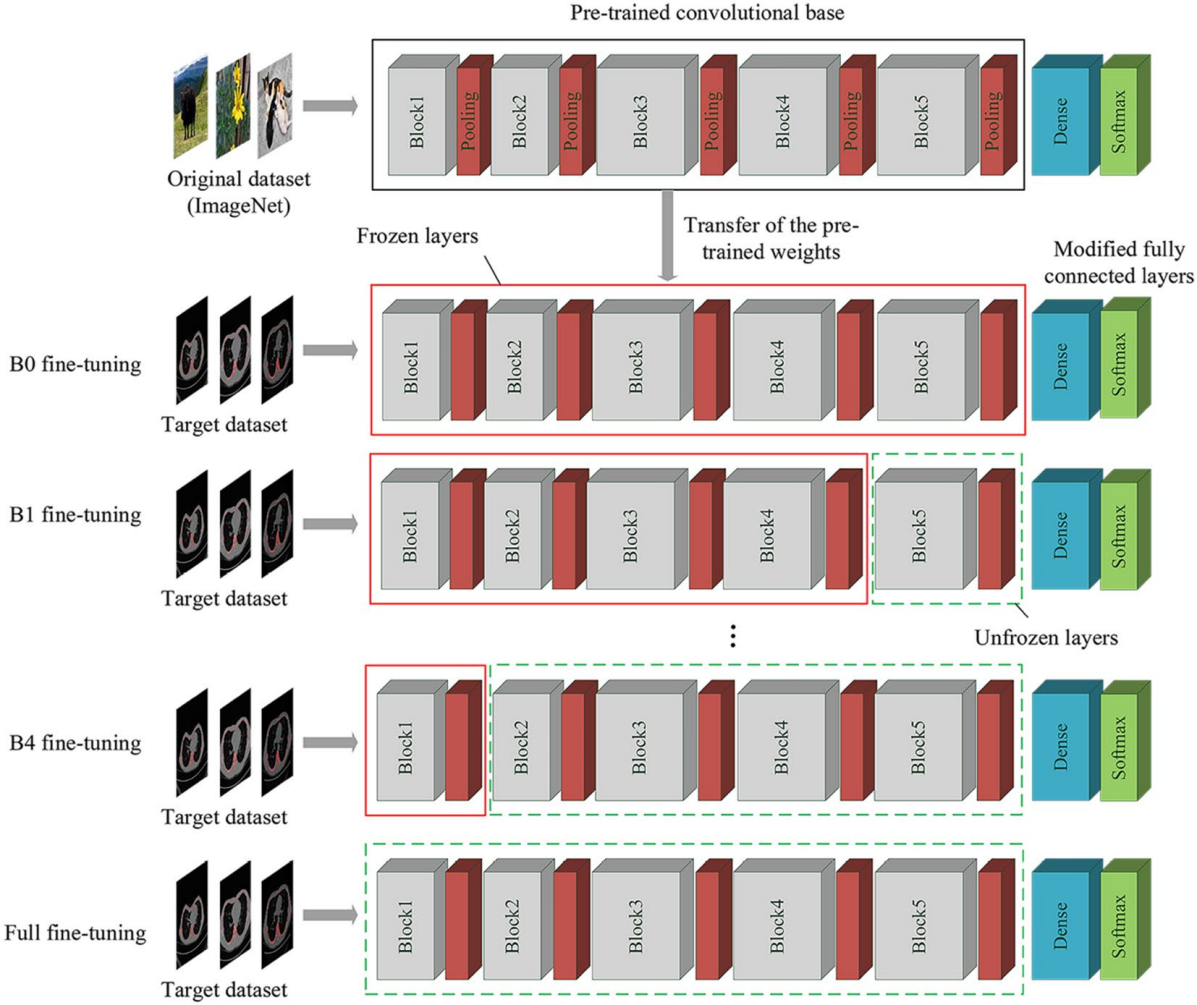
Block-wise fine-tuning process of the pretrained VGG network. Block1-Block5 represent the five convolutional blocks of the VGG network. Before fine-tuning, we modified the fully connected layers and built a new SoftMax classifier with three outputs for three categories: normal, mild and severe. B0 fine-tuning means that we freeze all the convolutional blocks and only train the new fully connected layers. Then, we gradually unfreeze the convolutional blocks until reaching Block 1. B*i* (where *i* is equal to 1, 2, 3, or 4) fine-tuning means that we freeze all the pretrained architecture except for the last *i* block and fully connected layers. Full fine-tuning means that we retrain all the convolutional blocks and fully connected layers.

Finally, after the fully connected layers were trained, we adopt a fine-tune strategy, that is, we unfroze the convolutional base block-wise, trained the model with a very small learning rate and continuously monitored the performance. In a CNN, the closer the network layer is to the top, the more abstract the features extracted by the CNN. From the bottom layer to the top layer, the features extracted by the neural network undergo an in-depth abstraction process from edges, shapes, and patterns to targets. Lower layers refer to the generic features that are independent of the task while higher layers extract the problem-specific features that are closely dependent on the task. To obtain better classification results and explore the impact of block-by-block fine-tuning on the model’s performance, we unfroze the convolutional base block-wise and resumed the backpropagation on the unfrozen layers. The block-wise fine-tuning process is shown in Fig. 4. Taking into account factors such as computing resources and time consumption, we adopt block-by-block fine-tuning instead of layer-by-layer fine-tuning.

#### Overall assessment of pectus excavatum

**Figure 5:**
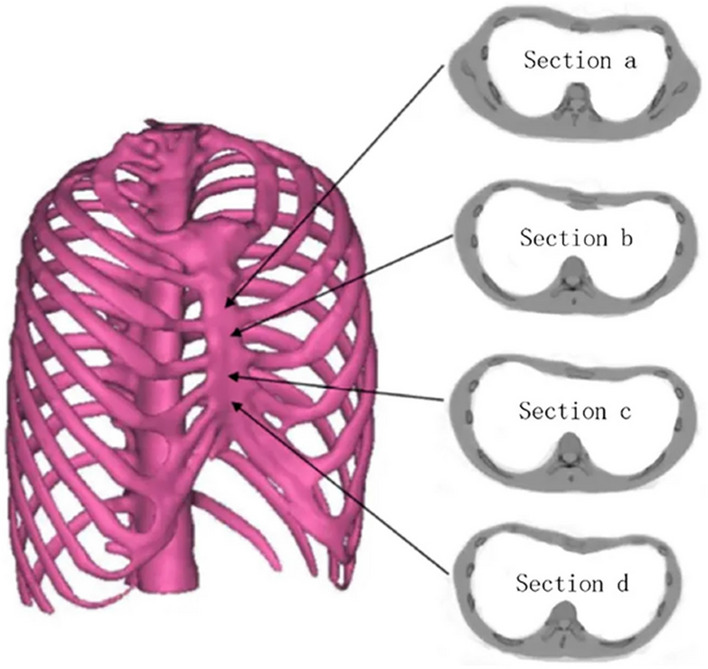
Schematic diagram of the reconstructed model of a patient with PE. The chest contours in different sections of the sternum have different degrees of depression. Reprinted with permission from Xie et al.^26^.

Based on the automatic assessment of each image, we further investigated the feasibility of comprehensive diagnosis for patients with PE. As shown in Fig. 5, the outline of the patient’s chest various from top to bottom and the cross sections of different parts have different shapes. Therefore, 2D images are not sufficient to document the deformities of the whole chest. It is necessary to take all images across the chest into account to provide a composite assessment of PE severity.

To address the limitation of 2D image-based diagnosis, we built a CAD system based on the proposed CNN model to provide an overall assessment of PE for each patient. First, the CAD system received and processed all images of each patient, and then automatically classify each image using the proposed CNN model. Second, the CAD system counted the number of images of each type, including normal, mild PE and sever PE, and output the proportion, respectively. The distribution of all CT images can reflect 3D components of the deformity of chest with PE. Finally, we adopted the majority rule-based voting strategy to draw a final comprehensive conclusion for the severity of each patient’s PE deformities. In brief, the proposed CNN classifier predicts the category label of each image, and then the majority rule-based voting method outputs the final predicted label as the one that receives most of the votes across all categories. For instance, if a patient has the largest proportion of its CT images in category A, the CAD system will make a prediction that category A is the overall category label. This outcome can be used as a comparative assessment to assist radiologists to get a better understanding of the condition of the entire chest instead of a certain region.

#### Model training and evaluation

Considering the size of our dataset and the model selection issues, we adopted 10-fold cross validation to overcome the shortcomings of the data scale and make full use of the dataset to test the pros and cons of the algorithm and the performance of the model. As listed in Table 1, the dataset is composed of 3703 pictures from 42 patients. It is noted that images from the same patient have some correlation and thus cannot be divided into the training set and the test set at the same time; otherwise, the classification accuracy obtained will be overestimated. Therefore, we separated the dataset in patient-level rather than dividing the subsets randomly. Images from the same patient would only be grouped into one fold. As a result, there are no overlapping images between the subsets, which means that the images from the same patient will not appear simultaneously in the training and test sets. Most of the subsets are typical representatives of the original dataset, and the proportion of each category remains within a non-extreme range. One subset is used as the test set while other nine subsets are sequentially used for training the CNN. We repeat this process to get ten models, and each sample in the dataset is used in the hold out set one time and used to train the model nine times. The average classification accuracy of the ten models on their respective test sets is used as the performance index of the classifier under this cross validation and can well reflect the generalization ability of the model.

Before training the newly added fully connected layers and conducting fine-tuning, we added a dropout layer to the dense fully connected layers so that some neurons in the hidden layer are randomly inactivated with a probability of 0.5, and the weights of these neurons are no longer updated during back propagation. The batch size we set is 64 and the SGD optimizer with a small learning rate is used when fine-tuning the network. We also adopted the early stopping strategy to continuously monitor the performance and reduce the risk of overfitting. The learning rate was initially set to 0.001 and would automatically decrease when the model training reaches a plateau. The training would end when the performance no longer improved. We implemented the experiments with PyCharm (using the Keras library with TensorFlow as a backend) and an NVIDIA TITAN Xp GPU with 12 GB of onboard memory.

### Ethics declarations

Ethics approval and consent to participate (i.e., informed consent) was obtained from all the participants to complete the protocol approved by the Guangzhou First People’s Hospital Department of Ethics Committee. Informed consent was obtained from the subject’s parent, if subjects are under 18. All the research was performed in accordance with the Declaration of Helsinki.

## Results

This section provides the validation results. First, we performed a comparison between the random initialization of the network and two transfer learning methods. Second, the effect of block-wise fine-tuning on the model’s performance were presented. Third, we visualized the areas activated by VGG network for each category. Finally, we showed the comprehensive diagnosis results obtained by our CAD system with the optimal configuration.

### Comparison of random initialization and transfer learning

In this part, we compared the random initialization of the network with two transfer learning methods to explore the impacts of different strategies on the model’s performance. Random initialization means that the neural network is trained from scratch with our dataset. We adopted the Gaussian distribution with a low deviation to initialize our weights to help the model converge to a meaningful optimal solution and overcome the negative effects of zero initialization. As an alternative, we used the pretrained VGG16 and VGG19 to extract the features of our dataset and saved the feature vectors as hdf5 files. We removed the top layers of the network (including the fully connected layers) and used the output of the last pooling layer of the VGG network as the feature vector. The output of the last pooling layer is $512\times 7\times 7$, which can be reshaped into a feature vector with a size of 25088 when flattening. A number of normalized features were selected through PCA method and the feature vectors were used to train three kinds of classifiers: an SVM, an LR and a DT. We used the GridSearchCV method to systematically traverse multiple parameter combinations and determine the parameters with the best effect through cross-validation. When training the SVM classifier, we used a linear kernel and set the penalty coefficient C to several specific values from 0.0001 to 1000. Similarly, we chose the appropriate max depth of the DT classifier and set the C parameter of the LR classifier via grid search. Finally, we used the pretrained VGG models and transferred the weights trained on ImageNet to our customized model, and then adopted the b0 fine-tuning method which means that we retained all the weights of the convolutional layers and only trained the fully connected layers.

The results of three methods are shown in Table 2. We can see that b0 fine-tuning method achieved highest performances, while the random initialization method performed well and the newly trained classifiers worked worst. On the one hand, features extracted with pretrained weights are not highly relevant to our task and are unsuitable for directly training new classifiers because the CT images differ greatly from natural images. Moreover, it is not appropriate for non-neural network (SVM, LR, DT) classifiers to handle such a large number of features. On the other hand, CNN models, which in our case is the random initialization and b0 fine-tuning method, had better classification performance on our dataset, both exceeding 83%. In addition, b0 fine-tuning improved accuracy by approximately 2–4% over random initialization. Our assumption is that compared with the randomly initialized weights, the transferred weights help models capture general features (such as edges) and converge to better results. The result inspires us that the fine-tuning strategy based on the transfer learning strategy outperformed the other methods with a highest accuracy of 88.38%, but it is still unsatisfactory if it is applied to medical diagnosis classification. In the next part, we will fine-tune the pretrained network block by block to achieve higher accuracy.

### Effect of block-wise fine-tuning

In this part, we explored the effect of block-wise fine-tuning on the model’s performance and investigated the degree of fine-tuning required to achieve optimal performance. We tested six fine-tuning schemes. B*i* (where *i* is equal to 1, 2, 3, or 4) fine-tuning means that we froze the entire pretrained architecture except for the last *i* convolutional block and fully connected layers. B0 fine-tuning means that we kept all the convolutional blocks frozen and only trained the new fully connected layers while full fine-tuning means that we unfroze all the convolutional blocks and updated the weights on the whole network.

**Figure 6:**
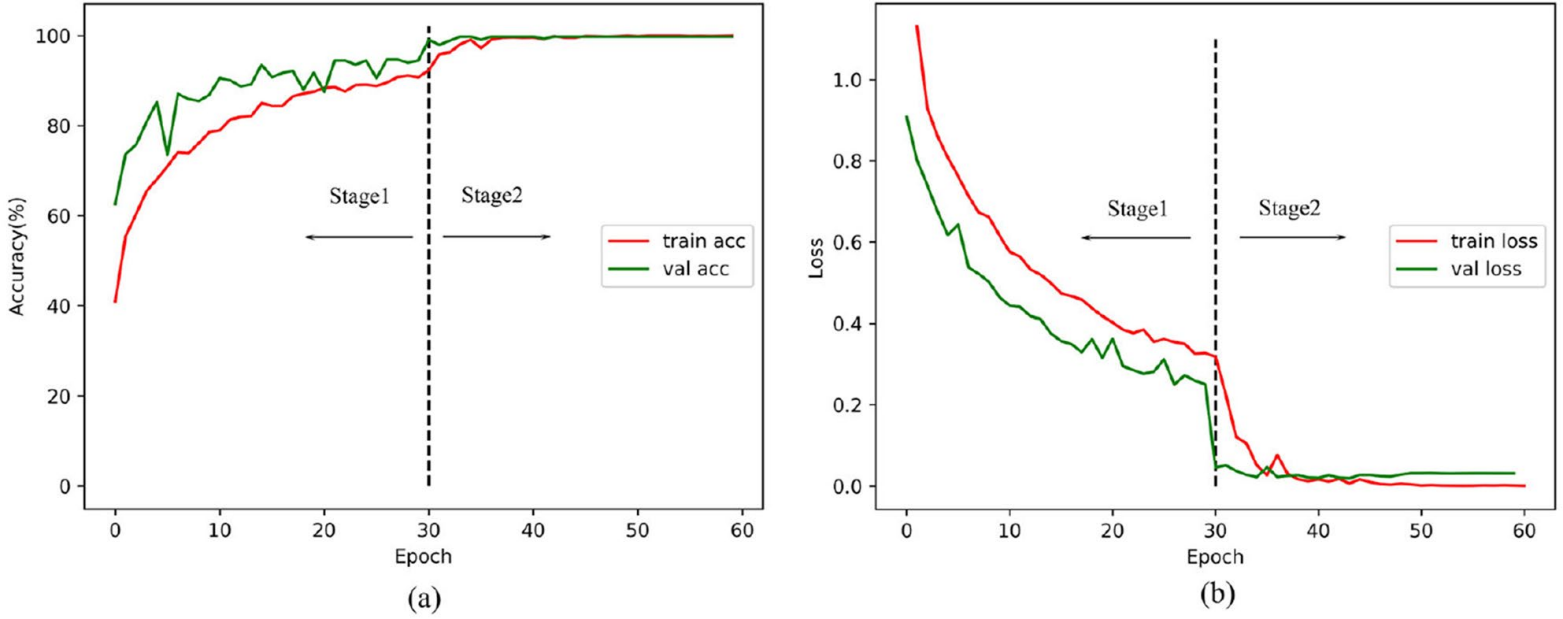
The training plots of accuracy and loss over the epochs for the pretrained VGG19 model using B2 fine-tuning method: (**a**) Accuracy graph of VGG19 model, and (**b**) Loss graph of VGG19 model.

Specifically, we monitored the performance of each model when performing fine-tuning. We randomly spilt the training set and used 15% of the data as the validation set, and the remaining 85% is used for training. The validation set was separated from the training set and used to monitor and tune the hyper-parameters. Figure 6 illustrates the training plots of accuracy and loss over the epochs for the pretrained VGG19 model using B2 fine-tuning method. As mentioned above, the fine-tuning went through two stages. We first froze all the convolutional layers of the pretrained network and only trained the newly added fully connected layers. Then, we started to unfreeze some of the convolutional layers and trained the network with a small learning rate. As shown in Fig. 6, there is a high degree of consistency between the training-validation accuracy and loss. The training would end when the performance no longer improved within ten epochs.

**Figure 7:**
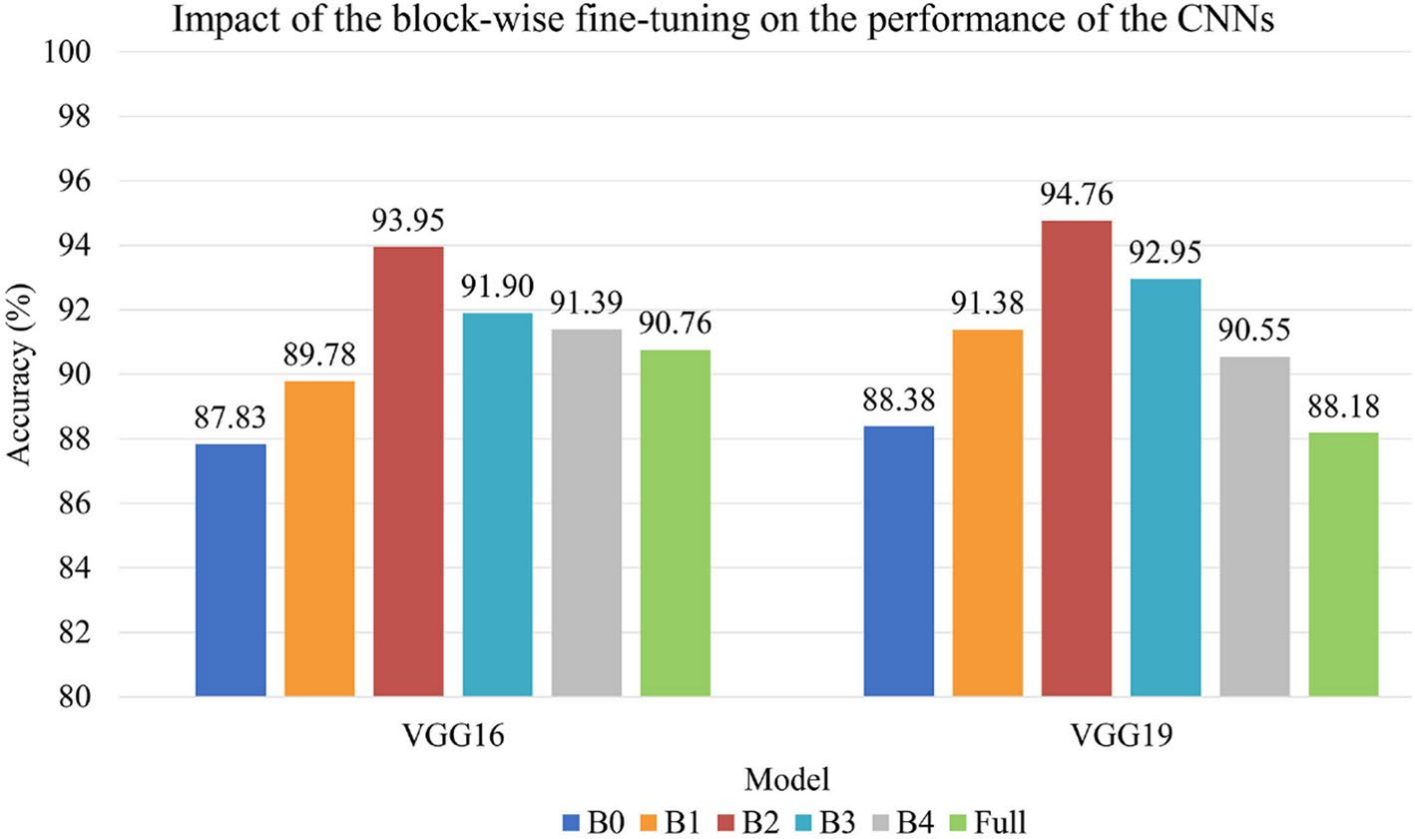
Comparison of the classification results obtained by different fine-tuning strategies (b0, b1, b2, b3, b4 and full fine-tuning).

The block-wise fine-tuning results are shown in Fig. 7. We can see that in VGG16 and VGG19 pretrained networks, b0 fine-tuning achieved the lowest classification accuracy compared with other fine-tuning schemes. The possible reason is that the network completely retains all the weights of the convolutional layers and replicates its capacities to learn the features of natural images instead of medical images. As the fine-tuning goes deeper by block, the classification accuracy of the model first becomes higher, and then gradually decreases after reaching a peak. The accuracy improves when the fine-tuning gradually deepens from b0 fine-tuning to b1 fine-tuning and b1 fine-tuning to b2 fine-tuning. Our assumption is that when we unfroze the last one or two convolutional blocks that are highly related to original dataset, the neural network will discard some features of the original dataset and start learning the problem-specific features that are closely dependent on our task. However, when the fine-tuning goes deeper into the bottom of the network, back propagation will occur between most layers and wreck the pretrained weights of shallow layers that are related to common features. Trained with a small-size dataset, a deep CNN replicated too many insignificant features from the training set and started overfitting. Therefore, models using b3, b4 and full fine-tuning methods performed worse compared with b2 fine-tuning schemes.

It is also noted that the VGG16 and VGG19 networks pretrained with the fine-tuning strategy achieved the highest classification accuracy of 93.95% and 94.76%, and they improved the accuracy by up to 10.15% and 8.79%, respectively, compared to random initialization. During the block-wise fine-tuning experiment, the optimal classification result, which indicates the optimal number of fine-tuned convolutional blocks, is achieved by the B2 fine-tuning method. Considering that the network with shallow fine-tuning has few unfrozen convolutional layers and trainable parameters to learn the typical features on the new dataset. And the weights of the model still too much involved the features of the original dataset. On the other hand, the deep fine-tuning would unnecessarily retrain the weights associated with the generic features at the bottom layers, resulting in too many training parameters for the model and thus overfitting on our small dataset^17^. Therefore, the optimal degree of fine-tuning should be balanced between shallow (too few training parameters) and deep fine-tuning (too many training parameters), which in our case is the B2 fine-tuning.

**Table 2:** The classification accuracies of three methods.

Model	Newly trained classifiers			Randomly initialization	B0 fine-tuning
	LR	DT	SVM		
VGG16	77.83	64.94	75.97	83.80	87.83
VGG19	79.88	61.82	80.31	85.97	88.38

A comparison between random initialization and two transfer learning methods is performed. The feature vectors extracted from our dataset with the pretrained VGG16 and VGG19 networks were used to train three kinds of classifiers: a logistic regression (LR), a decision tree (DT) and a support vector machine (SVM). B0 fine-tuning means that we retained all the weights of the convolutional layers and only trained the fully connected layers.

### Gradient-weighted class activation map (Grad-CAM)

**Figure 8:**
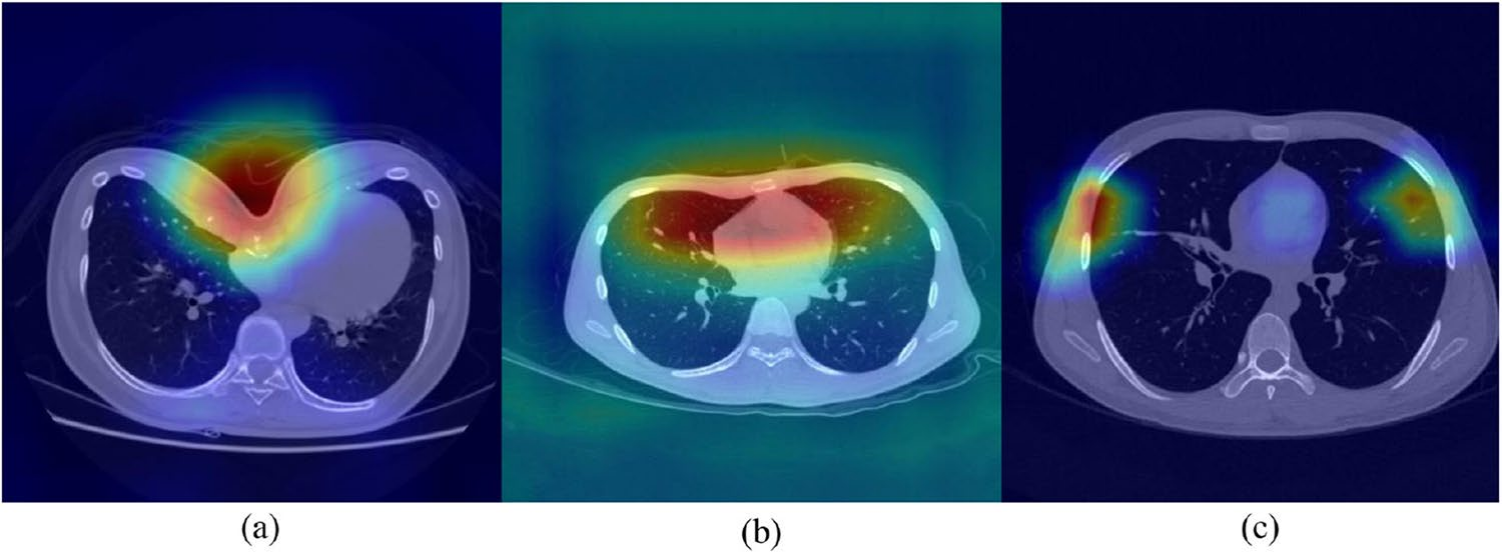
The Grad-CAM maps of three kinds of chest shape: (**a**) severe PE, (**b**) mild PE and (**c**) normal.

To understand what features have been learned and which area in the input images have been activated by VGG network, we used gradient-weighted class activation mapping (Grad-CAM) to extract gradients from VGG19’s final convolutional layer and highlight the regions that are most responsible for the prediction^27^. Generally, the red regions on the Grad-CAM map represent the areas where the network pays the most attention during the classification, while the blue regions receive the least attention. Several Grad-CAM maps of the three kinds of subjects are shown in Fig. 8. In the chest with severe PE, the salient areas of the Grad-CAM maps are located on the funnel-like depression of the anterior thoracic wall. For the cases with mild PE, the highlighted regions are still on the anterior thoracic wall, but not just the concave region but the wider areas. In normal cases, the network no longer focuses on the anterior chest wall. The Grad-CAM maps indicate that the VGG network utilizes features extracted from specific regions in the input images and draws corresponding classification conclusions.

### Comprehensive diagnosis results

**Figure 9:**
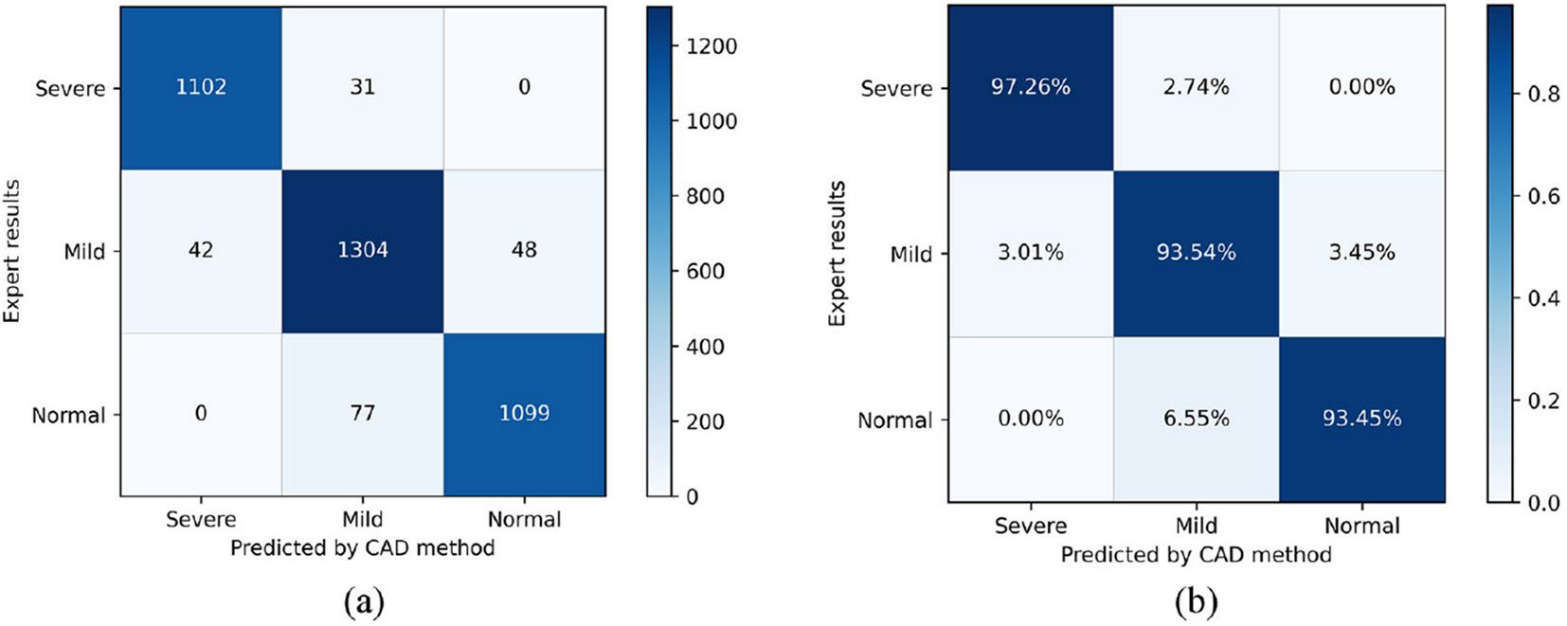
Confusion matrices of PE classification using the CAD system. (**a**) The number of observations in the confusion matrix. (**b**) The percentage of observations in the confusion matrix.

According to aforementioned analytical results, the best classification performance was achieved by adopting the b2 fine-tuning method and the VGG19 pretrained network. The confusion matrices of the entire dataset under this setup and 10-fold cross-validation is shown in Fig. 9. Our method has achieved a high level of performance in all three symptoms, with classification accuracy of 97.26%, 93.54% and 93.45%, respectively. These results indicated that our proposed model can accurately identify most images with slight errors in distinguishing similar symptoms (between severe and mild and between mild and normal).

After the proposed CNN classifier predicts the category label of each image, our CAD system integrated the classified results of all images for each patient, counted the percentage of all three symptoms and gave a final result based on the majority rule. We then compared the results obtained by the majority rule-based voting method in 42 cases with the diagnostic results given by radiologists. The confusion matrix of the comparison results is shown in Table 3. We can see that our CAD system has a reliable prediction for most patients, and only a small amount of uncertainty lies in the misdiagnosis of normal condition with mild symptom. We checked the misdiagnosed patient and found that the images of this patient are evenly distributed between the two categories of mild and normal. When a few images are misclassified (here some normal images are misclassified as mild symptoms), the final result derived from the majority rule may be biased. In general, our computer-aided diagnostic results are highly consistent with those given by radiologists with a consistency of 97.62% (the same diagnostic conclusion was obtained in 41 out of 42 cases). The differences in the comprehensive diagnosis results obtained by the CNN-based CAD system between different groups were assessed using nonparametric Mann–Whitney U test. Statistical analysis was performed using IBM SPSS statistics software (ver. 24.0, IBM Corp., Armonk, NY, USA), and a level of significance of 0.05 was selected. The average diagnosis performance of male subjects (95.03%) was better than female subjects (94.10%), but the difference was not significant (Mann–Whitney U test; P=0.651). The CNN-based CAD system showed a better performance in patients with severe PE with an average accuracy of 97.51%, followed by patients with mild PE with an average accuracy of 96.15% and subjects with normal chest with an average accuracy of 91.37%, but not significantly different.

**Figure 10:**
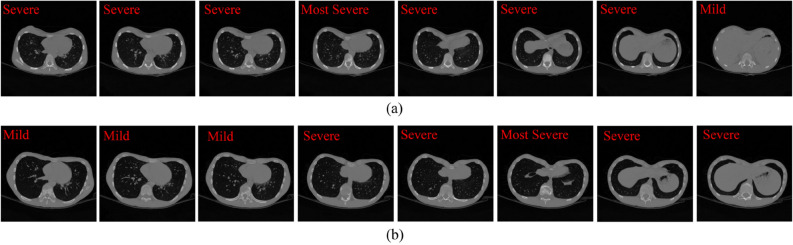
Several sample images of the two patients with PE. The images are selected from top to bottom at the same interval. All the images were labeled by experienced radiologists. (**a**) Several images of patient A. (**b**) Several images of patient B.

As mentioned above, a single image cannot not represent the overall characteristics of the patient. A series of CT images of two patients with PE are shown in Fig. 10. According to the HI assessment method, these two patients have approximately equal HI at the most severely depressed sternum contour. However, one typical image is not sufficient to document the deformities of the whole chest. When comparing a series of CT images, we can see that most parts of patient A are labeled as severe, while many parts of patient B are generally distributed between mild and severe. To make a comprehensively diagnostic evaluation, our proposed CAD system classified all images of these two patients, counted the number of images of each type, including normal, mild PE and sever PE, and output the proportion, as shown in Table 4. These two patients differ in the deformities of the whole chest. Patient A has a large proportion of severe CT images, while patient B has a much smaller proportion of severe CT images. These results provide additional diagnostic information, that is, although the most severe sternal depressions of patient A and B are similar, patient A is obviously in worse condition than patient B. The proportions output by our CAD system is highly consistent with the actual condition of the patients, which can reflect the overall severity of the patient. These comprehensive diagnosis results also can be employed as a reference for radiologists to evaluate the chest condition of patients and make a more appropriate treatment plan.

**Table 3:** Confusion matrices for all subjects between the overall results given by experts and the predicted results output by the CAD system.

Expert results	Predicted by CAD system		
	Severe	Mild	Normal
Severe	12	0	0
Mild	0	15	0
Normal	0	1	14

**Table 4:** A comparison of two patients with PE.

	Patient A			Patient B		
	Severe	Mild	Normal	Severe	Mild	Normal
Number of images	131	19	0	83	67	0
Percentage	87.33%	12.67%	0.00%	55.33%	44.67%	0.00%
Total numbers	150			150		
Haller index	4.47			4.44		

The Haller index in the table is calculated on the most severely depressed sternum of the two patients, and can represent their local severity.

## Discussion and conclusion

To the best of our knowledge, this is the first time that a CNN-based CAD system was applied for automatic assessment of PE. Our results indicated that the proposed CNN model with the transfer learning and fine-tuning strategy was able to classify the severity of PE deformities with an average high accuracy of 94.76%. By combining the classified results of various parts of the patient’s chest, our CAD system provided the proportion of images belonging to each symptom and drew comprehensive diagnosis results based on the majority rule, providing second objective opinions to assist radiologists in interpreting and diagnosing PE images. Comparing the results obtained by the majority rule-based voting system in the 42 cases with the diagnostic results given by radiologists based on clinical experience, we concluded that the proposed computer-aided diagnosis process has high reliability.

For automatic diagnosis of PE, many researchers were committed to modifying index-based methods to improve the evaluation metric and reflect the severity of PE deformities. Kim et al.^28^ proposed an algorithm related to CT image processing to automate the initialization and facilitate the index calculation process. Glinkowski et al.^29^ developed an optical 3-D chest measurement system and automatic data processing algorithms to assess the severity of deformities in PE. However, all these methods still relied on the subjective and manually selected indexes, which cannot represent the whole information of chest and be applied as a universal diagnostic and assessment approach due to the individual differences. To the best of our knowledge, previous works have not considered the combination of deep learning and a CAD system to eliminate subjective and manual feature extraction processes. Our results indicated the feasibility of using a CNN-based CAD system to intelligently and automatically learn discriminative features and evaluate the severity of a PE deformity.

In medical domains, a large amount of labeled data is usually unavailable, which is not conducive to the training of CNN. To address the overfitting problem caused by limited data, we adopted transfer learning strategy to improve the classification performance of the CNN model on our dataset. Our experimental results suggested that fine-tuning achieved better classification performance on our dataset than the feature extractor method for the application of transfer learning. Implementing the block-wise fine-tuning strategy on the pretrained networks can find the optimal fine-tuning degree and obtain the highest classification accuracy for the experiment. We adopted the b2 fine-tuning method, which means all the pretrained network layers were frozen except for the last two convolutional blocks to achieve the highest classification accuracies of 93.95% and 94.76% using the pretrained VGG16 and VGG19 networks, respectively. A possible explanation for this is that among the transferred weights, it is the weights of the bottom layer that are truly valuable for the new dataset because they represent the universal features in various images, including natural and medical images. The weights of the top layers that are related to the unique features of respective datasets must be retrained to fit our own dataset. This explains why modest fine-tuning works best.

Compared with the diagnosis based on a single image of a patient, our study considered the role of a single image classification in the overall diagnosis of a patient. Because the classification of a single image helped the overall diagnosis of the patient, we also focused on the comprehensive diagnosis of the patient as an individual. The proportion results given by our CAD system can well reflect the overall severity of PE deformities. We also followed the majority rule to draw a conclusion on the overall severity of patients, which is equivalent to assigning the same weight to each picture and then the category with the largest proportion can determine the overall characteristic of the patient. In fact, the same weight took into account the distribution of all image classification results for each patient on the basis of a certain rationality. We tended to think that based on what we have learned from our dataset, the same weight setting works well when using other new data.

The present study had several limitations. First, although we addressed the problem of limited annotated data using a data augmentation technique and transfer learning strategy, a larger sample of patients with PE is needed to draw stronger conclusions. Second, all images from the same patient were assigned the same weight and then combined to make a comprehensive diagnosis decision in this study. Considering that the presence of an image representing a severe symptom indicates more warning meaning than a normal image, the former may be given a higher weight and future studies should investigate the optimization of the weights to obtain more reasonable final results. Finally, our study is a preliminary attempt to apply deep learning to PE assessment. An automatic and application-oriented CAD system remains to be developed in further work.

This study had a number of strengths. It is the first time that a CNN-based CAD system was investigated for automatic diagnosis of PE. We exploited a CNN approach based on transfer learning and a fine-tuning strategy to classify the severity of PE deformities in various parts of each patient. We subsequently combined the classified results of various parts of the patient’s chest and derived the final diagnosis decision on the overall condition for each patient via majority rule. Our system eliminated subjective and manual processes and provided accurate and intelligent diagnostic advice.
